# Oxidative Stress and Intracranial Hypertension after Aneurysmal Subarachnoid Hemorrhage

**DOI:** 10.3390/antiox11122423

**Published:** 2022-12-08

**Authors:** Guangshan Hao, Pinar Eser, Jun Mo

**Affiliations:** 1 Department of Neurosurgery, The Fourth Affiliated Hospital, Zhejiang University School of Medicine, Yiwu 322000, China; 2 Department of Neurosurgery, Liaocheng People’s Hospital, Liaocheng 252000, China; 3 Department of Neurosurgery, Bursa Uludag University School of Medicine, Bursa 16059, Turkey; 4 International Institutes of Medicine, The Fourth Affiliated Hospital of Zhejiang University School of Medicine, Yiwu 322000, China

**Keywords:** subarachnoid hemorrhage, oxidative stress, intracranial hypertension, brain injury, antioxidant

## Abstract

Intracranial hypertension is a common phenomenon in patients with aneurysmal subarachnoid hemorrhage (aSAH). Elevated intracranial pressure (ICP) plays an important role in early brain injuries and is associated with unfavorable outcomes. Despite advances in the management of aSAH, there is no consensus about the mechanisms involved in ICP increases after aSAH. Recently, a growing body of evidence suggests that oxidative stress (OS) may play a crucial role in physio-pathological changes following aSAH, which may also contribute to increased ICP. Herein, we discuss a potential relation between increased ICP and OS, and resultantly propose antioxidant mechanisms as a potential therapeutic strategy for the treatment of ICP elevation following aSAH.

## 1. Introduction

Aneurysmal subarachnoid hemorrhage (aSAH) is a devastating neurological disease associated with unfavorable outcome and significant mortality as high as 45% of the cases, especially in young adults [1,2,3]. Unfortunately, recent developments in the diagnostics as well as the treatment of modalities have failed to arrive to an outstanding improvement in the functional outcomes of patients with aSAH [4,5].

Intracranial hypertension is very common in patients with aSAH, particularly in those with high-grade hemorrhages, in whom high intracranial pressure can lead to a vicious cycle that can be life-threatening if not treated in time [6]. However, the pathophysiological mechanism of intracranial hypertension after aSAH is complex and has not been fully clarified yet. Currently, there is no consensus guideline for the management of intracranial hypertension after aSAH.

In recent years, a growing body of evidence has suggested that oxidative stress may play a crucial role in pathological changes following aSAH [7,8,9]. Early brain injuries (EBIs), including the disruption of the blood–brain barrier (BBB), cerebral edema, and impaired cerebrovascular autoregulation, are thought to be responsible for elevated intracranial pressure after aSAH [10,11,12,13]. Since these pathological changes are significantly associated with oxidative stress, antioxidants may be potential candidates to treat intracranial hypertension after aSAH. Therefore, in this work, we review the mechanisms of OS and intracranial hypertension and their relationships, and propose antioxidants as potential therapeutic strategies for increased ICP after aSAH.

## 2. Characteristics of ICP in aSAH

The rupture of an intracranial aneurysm causes blood spread into the subarachnoid space, resulting with a sharp increase in the ICP, which can even transcend the average arterial blood pressure (MABP). In fact, there are several factors contributing to the elevated ICP after aSAH, including the presence of extravasated blood in the subarachnoid space, intracerebral hematoma formation, hydrocephalus, cerebral edema, and venous drainage disorders. The typical pattern of ICP changes after aSAH includes a subsequent decrease in the ICP value to a steady state within a few minutes, which nevertheless remains significantly high above the baseline. This phenomenon of declining ICP is explained by the Monro–Kellie hypothesis: the absorption of cerebrospinal fluid into the veins, the transference from the skull to the spinal canal, and the displacement of some of the venous and arterial blood from the cranial cavity [14]. When these compensations are insufficient, aSAH can lead to elevated ICP and even brain herniation.

### 2.1. Peak of ICP

The initial elevation of ICP is considered as a protective mechanism that prevents aneurysm from rebleeding [15]. On the other hand, elevated ICP leads to decreased cerebral blood flow (CBF) and subsequent cerebral ischemia, leading to vascular-derived and cytotoxic cerebral edema, which in turn leads to further elevation of ICP [16,17]. Therefore, a vicious cycle that constantly causes severe damage to the brain is triggered.

The main reason for the rapid rise in ICP after aSAH is due to the mass effect caused by blood extravasation into the subarachnoid space and the decreased buffering capacity of the intracranial space. The alterations in the dynamics of the cerebrospinal fluid system as well as the presence of blood cells and proteins in the subarachnoid space leading to disturbed cerebrospinal fluid (CSF) circulation and increased outflow resistance significantly contribute to the ICP elevation following aSAH [18]. Similarly, cerebral congestion caused by the vasoparalysis of distal cerebral arterioles [19], acute cerebral edema, and venous drainage disorders can be counted among other pathophysiological mechanisms, inducing an initial sharp increase in ICP. Moreover, the increased ICP has been shown to activate the sympathetic nervous system, which can trigger inflammation and cause an imbalance between the vasodilative and contractile factors [20,21,22,23]. Interestingly, an imbalance of brain tissue electrolytes occurs right after aSAH and plays a critical role in disease progression [24,25], but its relationship with elevated ICP remains unclear. 

### 2.2. Steady State

The elevated ICP value almost always falls down over a few minutes reaching a steady state, the level of which is much lower than the peak level but significantly higher than the baseline level [26,27,28]. It is important to note that, although the ICP values at this stage are well below the peak, the occurrence of intracranial hypertension at this stage is common and associated with poor outcomes [6,29]. 

At this stage, the blood breaks into the subarachnoid space, which could block the arachnoid granules and narrow the CSF passages such as the midbrain aqueduct and foramen of Monroe. The subsequent hydrocephalus contributes to the increase in ICP levels and are reported in more than half of the patients with aSAH [30]. In addition, it has been found that cerebral edema is the main reason for the increase in ICP levels at this stage, both in animal studies and clinical practice [31,32]. The cerebral edema can be observed in gray and white matter, as well as the deep cerebral nuclei and sometimes in the parasagittal watershed areas [33], while the oxidative stress-induced disruption of the BBB and brain ischemia have been proposed as key mechanisms for the development of cerebral edema after aSAH.

## 3. ICP Monitoring in aSAH

Although a strong association between increased ICP and aSAH has been reported in both experimental and clinical studies, currently, there is a lack of specific recommendations regarding the indications for ICP monitoring in patients with aSAH. ICP monitoring can provide more timely and intensive treatment for patients with aSAH when it comes to preventing secondary brain injury. In an international multicenter observational study enrolled 521 patients with aSAH, Chiara Robba et al. found that ICP monitoring might be associated with lower 6-month mortality, particularly in more severe cases [34]. Of note, ICP monitoring was probably carried out in selected patients with highly severe aSAH in the reported studies. Nevertheless, future studies are warranted in order to compose new protocols for the indications of ICP monitoring in aSAH. 

## 4. ICP and Outcome

Elevated ICP, conventionally and somewhat arbitrarily defined as ICP above 20 mmHg, is a common phenomenon after aSAH and may contribute to clinical deterioration. Clinical studies have revealed higher mortality rates and poor neurological outcome in patients with elevated ICP after aSAH [6,35]. The association between elevated ICP after aSAH and poor prognosis has been mainly conducted in retrospective studies, and the interpretation of the results is controversial due to the lack of large prospective studies. Interestingly, the results of a prospective observational study, including a total of 116 aSAH patients by Zoerle et al. on the relationship between elevated ICP and clinical outcome, revealed that although intracranial hypertension was associated with increased mortality, it was not independently related to unfavorable outcomes [35,36]. In another prospective study, Magni et al. have employed pressure–time dose (PTDICP) to quantify the burden and the time above four predefined thresholds (15, 20, 25, and 30 mmHg), and the high levels of PTDICP with thresholds set at 20, 25, and 30 mmHg were found to be associated with higher mortality at discharge, while moderate PTDICP 30 was related with a poor 6-month outcome [37]. Similarly, a recent retrospective multicenter study by Giogia Carra et al. demonstrated that the ICP pressure–time burden (duration and intensity of episodes of intracranial hypertension) was independently associated with 12-month outcome [38]. In this study, the researcher employed an ICP “dose” instead of a particular ICP cutoff and found ICP pressure–time burden to be an independent predictor of functional outcomes. 

## 5. Oxidative Stress in aSAH

Oxidative stress refers to the imbalance between the production of reactive oxygen species (ROS) and defending antioxidant systems. Several sources for the excessive generation of oxidative stress after aSAH have been introduced so far, such as hemoglobin degradation, disrupted mitochondrial respiration, intracellular peroxidases pathways, and disrupted antioxidant systems [39]. 

### 5.1. Hemoglobin Degradation

Extracellular hemoglobin and its metabolites (hemoglobin–heme–iron axis) are the main sources of ROS during the pathophysiological process after aSAH. After hemolysis, tetrameric hemoglobin is released from red blood cells and it degrades gradually, producing toxic intermediates. In the ferrous (Fe^2+^) and trivalent (Fe^3+^) states, heme can react with hydrogen peroxide to generate hydroxyl radicals through the Fenton reaction, which can damage lipid membranes, leading to the production of lipid ROS, cell dysfunction, and even ferroptosis [7,40,41].

### 5.2. Disrupted Mitochondrial Respiration

During normal mitochondrial respiration, electron transfer is accompanied by electron leakage from the transport chain and subsequent reaction with O_2_ to produce superoxides. This free radical is usually scavenged by the catalyzing enzyme superoxide dismutase (SOD). Three distinct types of SOD isozymes have been identified in mammals: cytosolic copper zinc SOD (SOD1), mitochondrial manganese SOD (SOD2), and extracellular SOD (SOD3) [42]. SOD2, the main mitochondrial antioxidant factor and ROS scavenger, is localized to the mitochondrial matrix and has been found to be involved in mitochondrial O_2_^−^ to H_2_O_2_ conversion [43]. During an ischemic phase (e.g., the ischemic phase after aSAH), the mitochondria become a source of excess free radical production, and the antioxidant enzyme is unable to scavenge this free radical [44]. Therefore, mitochondrial dysfunction due to ischemic injury following aSAH can lead to the leakage of superoxide anions and the excessive production of ROS. Studies on mitochondria activities after aSAH have consistently found disrupted mitochondrial respiration favoring the production of ROS. Marzatico and Baena et al. have reported increased levels of state 4 mitochondrial respiration and decreased respiratory control ratios following aSAH in association with increased ROS production [45,46]. Moreover, the overproduction of ROS due to mitochondrial dysfunction has been shown to be a key mechanism for cognitive dysfunction and poor prognosis [47,48].

### 5.3. Intracellular Peroxidases Pathways

In addition to the hemoglobin and mitochondria, a number of other enzymatic pathways in association with the production of free radicals have been investigated so far. Among these, several pro-oxidant enzymatic pathways have been thought to be associated with the overproduction of free radicals, including NADPH oxidase (NOX), myeloperoxidase (MPO), and nitric oxide synthase (NOS) [7,49,50,51,52].

The NOX family is a well-known and important source of ROS which is widely expressed in the central nervous system (CNS) cells except the oligodendrocytes [53,54]. The NOX catalyzes the transfer of two electrons through the biofilm to produce superoxide anion O_2_^−^ by using intracellular NADPH as an electron donor and extracellular molecular oxygen as a receptor. Then, O_2_^−^ is progressively metabolized to H_2_O_2_ and∙OH. The two major subtypes in the brain, NOX2 and NOX4, have been shown to significantly increase in the neurons as well as astrocytes around the hematomas of patients with aSAH [55]. Furthermore, a significant association has been shown between the NOX and delayed cerebrovascular spasm in experimental animal models, suggesting NOX as a potential risk factor for delayed ischemic neurologic deficit (DIND) after aSAH [56]. 

Myeloxidase (MPO) is a heme-containing peroxidase mainly found in the primary azurophilic granules of neutrophils and also in the primary lysosomes of monocytes in small amounts. After aSAH, the neutrophils are recruited in the subarachnoid space by the chemokines and produce large amounts of hypochlorous acid through MPO which in turn causes damage to the lipids, proteins, and DNA. MPO-mediated oxidative injury in CNS has been shown to cause cognitive impairment and neurodegeneration [57,58,59]. Consistent with these findings, clinical studies have revealed a positive correlation between the serum levels of MPO and the occurrence of DCI in patients with aSAH, and this has also been confirmed by experimental animal models [52]. 

The nitric oxide synthase (NOS) family consists of three subtypes: endothelial NOS (eNOS), neuronal NOS (nNOS), and inductive NOS (iNOS). The eNOS and nNOS are expressed constitutively, whereas the expression of iNOS requires a stimulation by the cytokines or other inflammatory products [60]. As the resident innate immune cells of the CNS, the microglia are initially activated following aSAH [61]. The microglia can then transform into the M1 phenotype and express the iNOS depending on the transcription factors, including hypoxia-inducible factor-1 and NF-κB. Subsequently, the iNOS increases the levels of NO, leading to free-radical-mediated neuronal damage [62,63]. 

### 5.4. Disrupted Antioxidant Systems

Nuclear factor erythroid-derived 2-related factor 2 (Nrf2), a major regulator of cellular antioxidant response, is widely expressed in the central nervous system and significantly upregulated in neurons, astrocytes, microglia, endothelin cells, and smooth muscle cells after SAH [64,65]. Nrf2 is a well-known redox-sensitive transcription factor; it is always located in the cytoplasm and is degraded by Kelch-Like Epichlorohydrin-Associated Protein 1 (Keap1). In response to the acute stress of SAH, Nrf2 is upregulated, released from Keap1 and transferred to nucleus, then bound to the antioxidant response element (ARE), which rapidly regulates the transcription of many detoxifying and antioxidant enzymes [65,66]. These include the catalase, SOD, glutathione reductase, and so on. Meanwhile, the activation of Keap1-Nrf2-ARE can also promote the degradation of erythrocytes and their degradation products through the upregulation of haptoglobin (Hp), hemopexin, HO-1, and ferritin [67,68,69,70]. However, these enzyme systems are disrupted and heavily consumed after aSAH, significantly reducing the antioxidant capacity of the brain tissue. In experimental animal models, aSAH has been shown to lead to a decrease in the activities of Zn and Cu-SOD, and studies of human aSAH have revealed a significant increase in the proportion of SOD/GSH-Px activity. In addition, endogenous antioxidant molecules such as glutathione, ascorbic acid, and tocopherols could be depleted after aSAH, which can be another cause of oxidative stress [7,8]. Moreover, the overexpression of Nrf2 and c-Jun could upregulate the ARE-mediated expression of gamma-glutamylcysteine synthetase (gamma-GCS), a scavenger of ROS [71]. However, studies on aSAH showed the deleterious effect of the JNK/c-Jun pathway due to pro-inflammatory effects [72,73].

**Figure 1:**
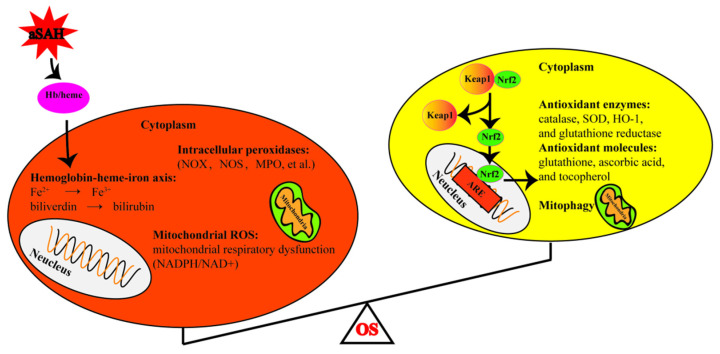
Schematic diagram of sources of oxidative stress and antioxidant system after subarachnoid hemorrhage. The left circle represents the production of the oxidants, while the right represents the antioxidants. OS: oxidative stress, aSAH: aneurysmal subarachnoid hemorrhage, Hb: hemoglobin, NADPH: nicotinamide adenine dinucleotide phosphate, NOX: NADPH oxidase, MPO: myeloperoxidase, NOS: nitric oxide synthase, SOD: superoxide dismutase. HO-1: heme oxygenase 1, Keap1: Kelch-Like Epichlorohydrin-Associated Protein 1, ARE: antioxidant responsive element, Nrf2: nuclear erythroid-related factor 2.

Mitophagy also plays an important role in the response to the OS after aSAH [74]. In response to mitochondrial stress after SAH, mitophagy could promote the clearance of irreversibly damaged mitochondria to encourage the survival of other mitochondria and thus the neurons. However, due to the severe injury stress, mitophagy is insufficient after SAH and neurons can inevitably undergo apoptosis [75] (Figure 1).

## 6. Oxidative Stress and Increased ICP

**Figure 2:**
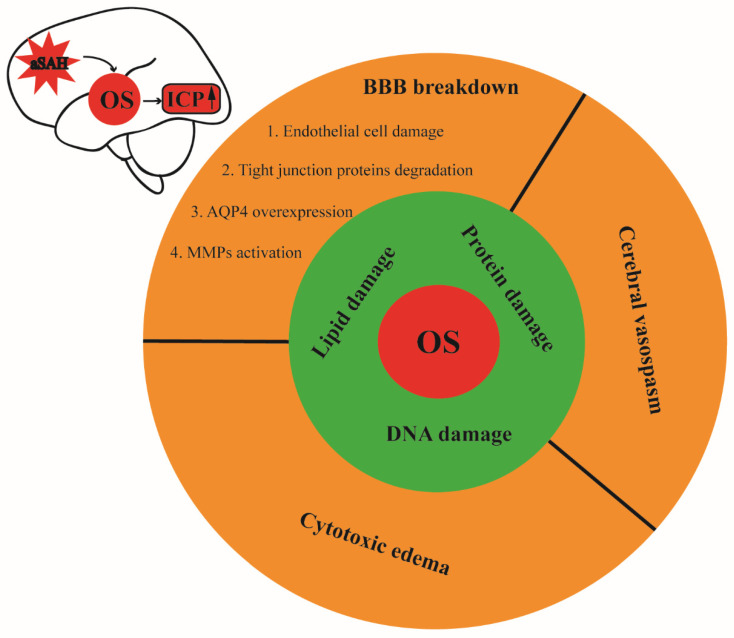
Schematic illustration of oxidative stress and its contribution to intracranial hypertension after aSAH. Oxidative damage to the lipids, proteins, and DNA can disrupt the BBB, vasoconstriction, and cytotoxic edema after SAH, leading to subsequent elevation of the intracranial pressure. OS: oxidative stress, ICP: intracranial pressure, aSAH: aneurysmal subarachnoid hemorrhage, BBB: blood–brain barrier.

Intracranial hypertension is a common complication of aSAH, which is also a critical determinator of the prognosis of patients with aSAH. Currently, there is evidence pointing to OS as a major contributor to increased ICP after aSAH. Importantly, impaired BBB is considered one of the most important causes of elevated ICP after aSAH. Specifically, OS can lead to the disruption of the BBB in several ways, including the apoptosis of the endothelial cells [10,12], damage to the tight-junction proteins [12], the activation of matrix metalloproteinases [10,12], and the elevated levels of aquaporin-4 [11] in patients with aSAH. Moreover, augmented levels of superoxide anions in the CSF after aSAH have been reported to display an association with cerebrovascular spasm, which may further lead to cerebral ischemia and elevated ICP [12,13]. OS-induced vasoconstriction is associated with NO depletion, the suppression of voltage-dependent K^+^ channels, and the upregulation of R-type Ca^2+^ channels in the cerebral arteries [62,76,77,78]. Moreover, OS-induced damage to the lipids, proteins, and DNA can lead to cytotoxic edema and the subsequent elevation of ICP as well (Figure 2).

## 7. Treatment

Intracranial hypertension is common among patients with aSAH. However, there are no specific treatment guidelines, and many current treatment recommendations are based on traumatic brain injury (TBI) guidelines. These recommendations include standard medical therapy (temperature control, head elevation, controlled hyperventilation with PaCO_2_ between 30 and 35 mmHg, osmotherapy, and CSF drainage), the surgical removal of space-occupying intracranial hematoma, and decompressive craniectomy [79,80,81,82]. Although patients potentially benefit from standard medical therapy, intracranial hypertension constitutes a major challenge when it is refractory [30]. We comparatively reviewed the two main treatment strategies (CSF draining and anti-OS therapy), and propose the best possible method in the management of increased ICP following aSAH.

Guidelines for the management of aSAH by AHA/ASA and the European Stroke Organization both recommend the use of CSF drainage to treat SAH-associated hydrocephalus [83,84]. The placement of an external ventricular drainage (EVD), continuous lumbar drainage, and lumbar puncture are the three commonly used strategies for CSF drainage in clinical practice. CSF drainage not only reduces intracranial pressure by alleviating hydrocephalus, but also allows the removal of accumulated intraventricular blood, thereby diminishing secondary damage to the brain tissue [85,86,87]. Consistently, decreasing elevated ICP via CSF drainage has been shown to correlate with improved cerebral microcirculation in patients with aSAH [88]. However, there is no consensus on the daily volume of required drainage, which is generally kept below 200 mL, adjusted according to the sign and symptoms of hydrocephalus or the ICP treatment threshold [89,90,91]. It should be noted that CSF over drainage and compensatory brain hyperemia can result in the malabsorption of CSF due to increased intracranial venous pressure and impaired CSF outflow. Therefore, impaired CSF circulation may lead to the aggravation of clinical symptoms and even coma, which can be reversed by immediately quitting the drainage [92]. The simultaneous monitoring of ICP and lumbar pressure was previously used as a treatment strategy to avoid lumbar over drainage by identifying progressive pressure gradients in advance [92]. With the development of technologies in new external drainage systems, it is now possible to regulate flow while monitoring ICP [93]. The system can detect the changes in the brain compliant by monitoring the ICP, thereby allowing the regulation of the CSF drainage based on the ICP levels. This avoids large fluctuations in the ICP and effectively prevents from excessive or insufficient drainage of the CSF. In fact, optimal CSF drainage should eliminate the blood degradations from the CSF circulation routes, and balance the formation and absorption of the CSF by ensuring proper brain compliance and cerebral blood supply.

During the last decade, the results of scientific research have shown that the genetic and pharmacological inhibition of OS alleviates multiple components of EBI, including elevated ICP. Hemoglobin is the main source of ROS after aSAH and has been shown as a potential therapeutic target. The physical clearance of blood either by surgically removing the hematoma or via CSF drainage has been proven to reduce the incidence of cerebral vasospasm, which might reduce intracranial hypertension [88,94]. Nrf2 is a transcription factor widely expressed in the CNS, and plays an important role in attenuating oxidative insults by regulating the expression of the genes involved in antioxidative response. Nrf2 has been shown to attenuate the disruption of the BBB, cerebral edema, and apoptosis through the Keap1-Nrf2-ARE pathway. Specifically, a variety of Nrf2 system activators, including the andrographolide, oleanolic acid, paeoniflorin, salvianolic acid A and B, aloperine, mangiferin, dimethylfumarate, astaxanthin, and L-cysteine, have been shown to significantly attenuate EBI after aSAH [95,96,97,98,99,100,101,102,103]. Similarly, docosahexaenoic acid, SS31 (a cell-permeable novel mitochondria-targeted peptide), Mdivi-1 (a selective Drp1 inhibitor), Mfn1-βIIPKC, fucoxanthin, bakuchiol, hydrogen, and metformin have been shown to have antioxidant stress and reduced cerebral edema after aSAH, and may be associated with its reduction in mitochondrial ROS [104,105,106,107,108,109,110,111,112]. The pharmacologic agents that potentially attenuate aSAH-induced OS and subsequent disruptions in the BBB and related pathways are summarized in Table 1 [113,114,115]. Since preclinical trials of several antioxidants mentioned above have shown them to protect the BBB and reduce brain edema, therapeutic strategies against ROS/OS may prevent ICP elevation after aSAH.

## 8. Conclusions and Future Directions

The mechanism of intracranial hypertension caused by aSAH is multifaceted and contributes significantly to the prognosis of the patient. Oxidative stress is an important pathophysiological mechanism that causes intracranial hypertension, rendering oxidative stress as a potential target for the treatment of intracranial hypertension. The complex network among oxidative stress, intracranial hypertension after aSAH, and the outcome is still unclear and requires further accurate identification. Thus, future research on the contribution of different pathways of oxidative stress to raised ICP after aSAH is of great importance. Preclinical trials of several antioxidants have been shown to protect the BBB, reduce brain edema, and improve prognosis in aSAH animal models. Thus, we propose antioxidant mechanisms as a potential therapeutic strategy for the treatment of ICP elevation after aSAH. However, future large randomized controlled trials are warranted to acquire a better understanding on the relationship among oxidative stress, raised ICP, and the clinical outcomes in patient with aSAH.

## Figures and Tables

**Table 1:** The results of experimental studies on antioxidant systems and intracranial hypertension following aSAH.

Anti-OS	Pathway	Medicine	Possible Effects of ICP	Effects of OS
Upregulate anti-OS system	Keap1-Nrf2-ARE	Andrographolide [103]	attenuate neuronal apoptosis, BBB disruption, and brain edema	
		Oleanolic acid [102]	reduce brain edema, BBB disruption, and neuronal apoptosis	increase the levels of superoxide dismutase, catalase, and GSH-Px
		Paeoniflorin [101]	attenuate brain water content, Evans blue extravasation, and neuronal apoptosis	decrease ROS, MDA, 3-nitrotyrosine, and 8-OHDG levels; increase SOD, GSH-Px, and CAT activity
		Salvianolic acid A and B [100]	reduce brain edema and neuronal apoptosis	suppress ROS; decrease lipid peroxidation; and increase GSH-Px, GSH, and SOD activities
		Aloperine [99]	ameliorate brain edema and cellular apoptosis	decrease MDA and increase GST
		Mangiferin [98]	ameliorate brain edema and cellular apoptosis	decrease MDA; increase SOD, CAT, and GSH
		Dimethylfumarate [97]	attenuate brain edema and BBB impairment	decrease MDA; increase SOD, NADPH NQO1, and GST-a1 activities
		Astaxanthin [96]	attenuate brain edema, BBB disruption, and cellular apoptosis	decrease MDA; increase NQO1 and GST-a1 activities
		L-cysteine [95]	decrease brain water content	reduce ROS content and decrease endoplasmic reticulum stress
Reduce ROS	Mitochondrial pathway	Docosahexaenoic acid [107]	ameliorate mitochondrial dysfunction, reduce brain edema, and attenuate OxyHb-induced neuronal death	attenuate MDA levels and SOD stress
		SS31 [106]	ameliorate mitochondrial dysfunction, brain edema, and Evans blue dye extravasation; decrease neuronal apoptosis	reduce MDA levels and restore the activities of GSH-Px and SOD
		Mdivi-1 [108] (a selective Drp1 inhibitor), dynamin-related protein-1 (Drp1, a dominator of mitochondrial fission)	ameliorate BBB disruption and brain edema, decrease the expression of MMP-9, and prevent the degradation of tight-junction proteins	reduce ROS levels
		Mdivi-1 [105]	attenuate the release of cytochrome C from mitochondria, inhibit excessive mitochondrial fission, restore the ultra-structure of mitochondria, alleviate brain edema and BBB permeability, and attenuate apoptotic cell death	reduce levels of MDA, 3-NT, and 8-OHdG; improve SOD activity
		Mfn1-βIIPKC [104]	attenuate the OxyHb-induced neuronal injury and apoptosis; reduce brain edema	enhance the activities of its downstream mitochondrial antioxidant enzymes
		Fucoxanthin [109]	improve mitochondrial morphology, ameliorate neural apoptosis, and reduce brain edema	decrease intracellular MDA, nitrotyrosine, and 8-OHDG production and increase endogenous antioxidant systems (including GSH-Px, GSH, SOD, and catalase)
		Bakuchiol [110]	alleviate BBB disruption (decrease EB extravasation; increase claudin-5, occludin, and zonula occludens-1; and decrease matrix metalloproteinase-9) and brain edema; inhibit cellular apoptosis by regulating the protein levels of Bcl-2, Bax, and cleaved caspase-3	attenuate oxidative stress by reducing reactive oxygen species, MDA, 3-NT, 8-OHDG, gp91 phox, and 4-HNE; increase the activities of SOD and GSH-Px
		Hydrogen [111]	reduce the expression of apoptotic makers in the vessels, brain edema, microthrombi formation, and vasospasm	decrease MDA concentration, 8-OHDG-positive cells, and the expression of 4-HNE and HO-1; increase SOD2
		Metformin [112]	attenuate brain edema and disrupt BBB permeability	alleviate OS
Other pathways	ER stress	Apelin-13 [113]	attenuate brain edema and preserve BBB integrity (Evans blue staining)	reduce MPO and ROS
	MAPK	Naringin [114]	ameliorate brain edema and BBB integrity	decrease MDA; increase the activities of CAT, GSH-Px enzymes, and the GSH/GSSG ratio
	Akt and NF-κB pathways	3,4-Dihydroxyphenylethanol [115]	induce a reduction in the brain water content and decrease BBB permeability	decrease MDA; augment the activities of SOD, CAT, and GSH-PX

8-OHDG: 8-hydroxy-2-deoxy guanosine, BBB: blood–brain barrier, CAT: catalase, GSH-Px: glutathione peroxidase, GST: glutathione S-transferase, GST-a1: glutathione S-transferase a1, MDA: malondialdehyde, MPO: myeloperoxidase, NADPH: nicotinamide-adenine dinucleotide, NQO1: quinone oxidoreductase 1, OS: oxidative stress, ROS: reactive oxygen species, SOD: superoxide dismutase.
